# Exploring the Effects of Tirzepatide on Obstructive Sleep Apnea: A Literature Review

**DOI:** 10.7759/cureus.80164

**Published:** 2025-03-06

**Authors:** Jimmy Wen, Denise Nadora, Alina Truong, Ethan Bernstein, Christiane How-Volkman, Daniel I Razick, Muzammil Akhtar, Adam A Razick, Eldo Frezza

**Affiliations:** 1 Physical Medicine and Rehabilitation, California Northstate University College of Medicine, Elk Grove, USA; 2 Internal Medicine, California Northstate University College of Medicine, Elk Grove, USA; 3 Orthopedic Surgery, California Northstate University College of Medicine, Elk Grove, USA; 4 College of Medicine, California Northstate University College of Medicine, Elk Grove, USA; 5 Surgery, California Northstate University College of Medicine, Elk Grove, USA; 6 Psychology, University of California Los Angeles, Los Angeles, USA

**Keywords:** glp-1 agonist, obstructive sleep apnea, osa, tirzepatide, weight loss

## Abstract

Obstructive sleep apnea (OSA) is a sleep disorder commonly secondary to obesity that has detrimental effects on health and quality of life. Thus, weight loss is one of the mainstays of OSA treatment. Tirzepatide, a novel glucagon-like peptide-1 (GLP-1) and glucagon-dependent insulinotropic polypeptide (GIP) dual agonist, demonstrated significant glycemic control and weight loss. This literature review analyzes the current literature on tirzepatide’s effects on OSA, mechanism of action, complications, and off-label uses/indications. Also, this review offers potential insights into how tirzepatide and other GLP-1 medications can be repurposed for other metabolic conditions and their associated sequelae.

## Introduction and background

Tirzepatide is a novel glucagon-like peptide-1 (GLP-1) and glucagon-dependent insulinotropic polypeptide (GIP) dual agonist medication that was approved by the US Food and Drug Administration (FDA) in May 2022 for type 2 diabetes mellitus (T2DM) and in November 2023 for weight loss for obesity [1]. Tirzepatide is available as a once-weekly subcutaneous injection at 2.5mg, 5mg, 7.5mg, 10mg, 12.5mg, and 15mg, with an initial dosage starting at 2.5mg and escalating as needed [2]. Its glycemic control and weight loss effects are dose-dependent and work similarly to other GLP-1 medications such as semaglutide and liraglutide. The SURPASS trials show that tirzepatide provides superior clinical glycemic control and weight loss compared to semaglutide, dulaglutide, insulin degludec, and insulin glargine [1]. Although GLP-1 agonists produce substantial weight loss, the effect on overall body composition is unclear. There appears to be a resulting decreased lean mass and fat-free mass, albeit not in a significant manner. The most commonly observed adverse effect is in the gastrointestinal system (nausea, vomiting, abdominal pain, and bloating). Additionally, delayed gastric emptying is also a recent concern as it can raise the risk of regurgitation and aspiration [2]. 

Obstructive sleep apnea (OSA) consists of episodic complete upper airway collapse (apnea) or partial collapse (hypopnea) with decreases in oxygen saturation, resulting in disturbed sleep [3]. Adverse effects of OSA include associated hypertension, hyperlipidemia, heart failure, arrhythmia, T2DM, and pulmonary hypertension [4]. Depending on the etiology of OSA, the short-term prognosis with treatment is favorable, but the long-term prognosis is variable. This is mainly due to the low rates of continuous positive airway pressure (CPAP) adherence, estimated at nearly 50% despite patient education. Many OSA patients have comorbidities that place them at higher risks for cardiovascular and cerebrovascular events in addition to the adverse events mentioned earlier [3]. Many contributory factors play a role in pharyngeal narrowing, notably large neck circumference commonly secondary to obesity, male sex, sedentary lifestyle, tobacco, and alcohol usage [5]. Obesity has become a major global health issue and is considered one of the main causes of OSA [6]. The prevalence of OSA is concurrently increasing, paralleling the prevalence of obesity across the globe, ranging from 14% to 55% [1]. Interestingly, the severity of OSA is inversely correlated with age when controlling for body mass index (BMI) [7]. In their case-control study, Patial et al. found that the majority of their OSA patients were secondary to obesity and diabetes-related conditions and suggested that pharmacological interventions hold promise for altering obesity-associated anthropometric variables to improve OSA symptoms [6]. Weight loss is one of the mainstays of OSA treatment and provides additional benefits by mitigating cardiovascular and metabolic risk factors and markers [4]. 

Ultimately, the treatment of OSA requires a multi-faceted but individualized approach for each patient. Bariatric surgery is an effective way to reduce and maintain weight loss, but it may not be feasible for every patient and comes with its own morbidity and perioperative risks [8]. GLP-1 receptor agonists (RAs) have well-documented effects on weight loss and glycemic control via delayed gastric emptying and reduced food intake by binding central receptors in the hypothalamus and throughout the central nervous system (CNS). Thus, the advent of this drug class has led to increased interest in using these medications for sequelae caused by obesity and T2DM. A 2024 scoping review by Le et al. found that GLP-1 RAs show early evidence of their positive effect on OSA by reducing the apnea-hypopnea index (AHI), a measure of the severity of OSA [9]. This literature review aims to synthesize the currently available literature surrounding the effects of tirzepatide on OSA, mechanisms of action, complications, and off-label uses/indications. Additionally, this review aims to provide potential insights into how tirzepatide and other GLP-1 RAs may be repurposed for other metabolic conditions and their associated sequelae. 

## Review

Effect on obstructive sleep apnea (SURMOUNT-OSA)

Malhotra et al. conducted two phase 3 controlled clinical trials to evaluate the effects of tirzepatide on OSA patients utilizing a double-blind, randomized research design [10]. After participants were separated into their respective trial groups, they were randomly assigned to either a weekly tirzepatide or placebo treatment for 52 weeks. In both conditions, participants received weekly doses subcutaneously. The participants of the study were adults aged 36-62 diagnosed with moderate-to-severe OSA and obesity. Trial 1 consisted of participants who did not use positive airway pressure (PAP) prior to baseline, while trial 2 consisted of participants who were utilizing PAP a minimum of three months prior to screening and planned continued use throughout the study [10]. 

The primary metric measured by the researchers was the change in the AHI over the 52 weeks. This statistic measures the number of apnea and hypopnea episodes during one hour of sleep. Secondary outcomes included percentage change in body weight, changes in systolic blood pressure, and patient-reported disturbance during sleep using the Patient-Reported Outcomes Measurement Information System (PROMIS). Tirzepatide was shown to be an effective treatment for participants in both trials. In trial 1, there were approximately 20 fewer events per hour on average for those in the experimental group versus those prescribed a placebo (P <0.001). In trial 2, there was an even greater mean reduction in events during sleep at 23.8 events per hour (P <0.001). Percent change in body weight was significant in both trials, with experimental patients in trial 1 demonstrating an average 17.7% reduction and patients in trial 2 demonstrating an average 19.6% reduction in body weight, compared to 1.6 and 2.3 percent in control groups in each trial, respectively. Current guidelines for OSA recommend a weight reduction of 7% to 11% for OSA patients, with a recent meta-analysis associating further weight reduction with a reduced AHI [10,11]. The mean change in the AHI was -25.3 and -29.3 events per hour in trials 1 and 2, respectively. Mean changes in systolic blood pressure were -9.5 and -7.6 mmHg in trials 1 and 2, respectively. Pooled mean changes in PROMIS sleep-related impairment (SRI) scores for both trials were -7.5 in the tirzepatide groups compared to -3.6 in the placebo groups. Pooled mean changes in PROMIS sleep disturbance (SD) scores for both trials were -5.7 in the tirzepatide groups compared to -2.7 in the placebo groups. Across both trials, up to 50.2% met the combined secondary end-point criteria of less than five AHI events per hour or 5 to 14 AHI events per hour and an Epworth Sleepiness Scale (ESS) of 10 or less [10]. This meaningful proportion of patients achieving these criteria is important as these thresholds represent disease severity at which PAP therapy may not be recommended. Additionally, symptom severity in OSA is associated with disease burden and increased risk of cardiovascular complications. Thus, the observed improvements in sleep function via PROMIS-SRI and PROMIS-SD are clinically relevant. 

Adverse effects were reported in 190 of 234 patients (81.2%) in the tirzepatide groups across both trials. However, 175 of 233 patients (75.1%) in the placebo groups also reported adverse effects. The most frequently reported complaints in the tirzepatide groups across both trials included diarrhea (23.9%), nausea (23.5%), constipation (15.4%), and vomiting (13.2%). There were zero cases of death across tirzepatide or placebo groups in either trial [10]. Therefore, this study provides evidence that tirzepatide can be an effective treatment for patients with moderate to severe OSA.

Additionally, a review of the SURPASS and SURMOUNT-1 studies conducted by Sinha et al. discussed the efficacy of tirzepatide as it relates to DM and obesity management. Obesity is closely linked to T2DM, as it causes insulin resistance in patients, with both conditions being commonly associated with many metabolic complications, including OSA [12]. Weekly tirzepatide doses of 5 to 15 mg in patients with T2DM demonstrated reductions in body weight ranging from 5.4 to 12.9 kilograms (kg) over periods of treatment up to 104 weeks. Moreover, significant improvements in cardiometabolic risk factors as well as reduced glycosylated hemoglobin (1.87% to 3.02%) were found in participants with T2DM throughout the clinical trials [12]. Compared to the average 6.2 kg weight reduction seen in the SURPASS-2 trial utilizing 1 mg of semaglutide, T2DM patients receiving tirzepatide doses of 5, 10, or 15 mg achieved average reductions in weight of 7.8, 10.3, and 12.4 kg, respectively [13]. Therefore, while semaglutide (Ozempic and Wegovy) has taken many of the headlines, tirzepatide (Zepbound and Mounjaro) may be the superior option in weight loss reduction and glycemic control. 

OSA epidemiology and risk factors

Understanding the patterns and factors that contribute to the pathophysiology of OSA provides insights that help determine treatment and preventative measures for this condition. However, there is great heterogeneity in the methodology of research studies that seek to understand this condition further. Therefore, the prevalence of OSA within the population samples is often not representative of the general population [14]. Despite these differences, most epidemiology studies use the AHI to diagnose OSA [15]. Using the AHI, several population-based studies have been conducted in different geographical regions and ethnic groups. Overall, the prevalence of OSA varies between 9% and 38% in the general adult population [16]. Interestingly, OSA is more prevalent in men than women. A study by Young et al., including 602 men and women, showed that OSA was prevalent in 9% of women and 24% of men [17]. Furthermore, in individuals older than 65 years old, 50% of the patients showed AHI > 5 and 20% showed AHI > 15 events per hour [18]. The widespread prevalence and demographic variation of OSA highlight its significant impact on public health.

OSA may affect any individual of all ages and gender. However, certain factors may increase the risk of developing OSA including obesity and a history of recreational drug use. OSA presents a bidirectional relationship with obesity, increasing the risk of developing OSA. A study by Phillips et al. showed that patients diagnosed with OSA recently experienced a significant weight gain in the year preceding the diagnosis [19]. On the other hand, in a mechanism poorly understood, OSA symptoms such as sleep fragmentation may also contribute to patients' rapid weight gain, promoting obesity in patients with OSA [20]. Additionally, it was found that recreational drug use such as smoking and alcohol increases the risk of OSA. In a study involving 57 subjects with OSA, it was found that moderate to severe OSA was more common in individuals who smoke, with a positive correlation between smoking duration and symptom severity [21]. Similarly, a meta-analysis of 21 studies showed an increased risk of OSA in individuals who consumed alcohol compared to those who did not, with a positive correlation between the quantity of alcohol consumed and the risk of OSA. Due to the multifactorial effect of alcohol in the body including predisposition to upper airway collapse and increased body mass index, increased alcohol intake has provided evidence of increasing the risk of OSA [22]. While these factors significantly contribute to the overall risk of developing OSA, other uncontrollable variables also warrant consideration.

Other risk factors that may be difficult to control and alter include family history and craniofacial abnormalities. Patients who have first-degree relatives of people with OSA were found to be more likely to develop OSA [23]. A genome-wide study involving 12,558 Hispanic American participants showed that breathing disturbances including apneas and hypopneas were associated with polymorphisms in the G-protein receptor gene 83, a gene expressed in several regions of the brain [24]. This illustrates that mutations in the genetic code may play a role in the development of OSA. Conversely, changes in the facial structures of various etiologies have also been associated with OSA. In a case-control study using magnetic resonance imaging cephalometry, two important factors were found to be significantly associated with OSA [25]. Firstly, a smaller mandible was a risk factor for men but not for women. Secondly, an inferior-posterior position of the hyoid bone was significantly associated with the risk of OSA in both males and females with an enlarged tongue [25]. Given the various factors that can play a role in increasing the risk of developing OSA, understanding these variables can help guide preventive measures and targeted interventions in patients with OSA.

Conventional treatment options for OSA and current challenges

Treatment options for OSA can be categorized into three main types: behavioral, medical device, and surgical. Recommendations vary depending on age and demographics.

Positive Airway Pressure Treatment

In adults, the most common non-invasive treatment is PAP, which includes CPAP and bilevel positive airway pressure (BiPAP). PAP machines provide pressurized air to maintain positive pharyngeal transmural pressure, counteract surrounding pressure, and prevent nocturnal collapse. This treatment has been shown to decrease the severity of symptoms of OSA, including reductions in daytime fatigue and hypopnea frequency [26]. Studies have also demonstrated a reduction in OSA severity and overall improvement in quality of life [27]. Additional benefits include moderate improvement in hypertension, particularly in patients with resistant hypertension, a lower risk of crashes of energy throughout the day, better glycemic control in diabetic patients, and enhanced overall quality of life. Some studies suggest that OSA treatment could decrease the frequency of cardiovascular events, although this depends on adequate treatment adherence [28].

The main challenge associated with PAP treatment is adherence. Patients often find the device cumbersome and unsightly. Studies on CPAP non-compliance have shown that cost and mask discomfort are highly correlated with non-compliance, while health coverage has a positive predictive value for compliance [29]. Other predictors of non-compliance include the spring and summer months, leakage around the mask, advanced age, prior uvulopalatopharyngoplasty (UPPP), and lower mean oxygen desaturation index, resulting in mild symptoms [30,31]. Patients who had used the PAP mask for ≥4 years were more likely to be adherent, indicating that non-compliance is more common early in treatment. Non-compliance is also associated with higher variability in sleep onset and irregular circadian rhythms compared to adherent patients [32].

Behavioral Treatments

Behavioral treatments aim to reduce risk factors associated with nocturnal airway collapse. Lifestyle interventions targeting weight reduction, such as diet modification and exercise, are mainstays in treatment and are recommended alongside CPAP usage. Exercise, in addition to aiding weight loss, can independently improve the AHI, quality of life, sleep quality, and exercise capacity in OSA patients [33]. Modifying sleeping positions has also shown positive results, with Benoist et al. demonstrating similar outcomes in non-supine sleeping positions compared to oral appliance therapy [34].

Oral Devices

Oral devices, such as mandibular repositioning devices, reduce OSA severity by protruding the mandible anteriorly and altering jaw and tongue positions to prevent airway collapse [35]. Although these devices are less effective than PAP machines, they have higher compliance rates and are established as second-line treatments for medical devices [36].

Surgical Treatments

Most surgical treatments aim to stabilize the airway and prevent nocturnal collapse. UPPP, which involves resection of the uvula and partial resection of the soft palate and lateral pharyngeal walls, has shown good results but lower efficacy than PAP [37]. Other surgical options include maxillomandibular advancement, tracheostomy, and hypoglossal nerve stimulation, which stabilize the airway effectively but are less common due to longer recovery times, invasiveness, and patient preference [38]. Bariatric surgery is another option, which can improve OSA symptoms secondary to weight loss. 

Pediatric Treatments

In pediatric patients, the most common form of OSA is adenotonsillar hypertrophy, though obesity is also a possible cause [39]. Treatment is crucial due to risks of failure to thrive, inattention, behavioral problems, and early onset cardiovascular issues. Surgical options include adenotonsillectomy, with PAP recommended if surgery is unsuccessful [40]. Rapid palatal expansion, taking advantage of the patient’s growth potential and patent palatine bone, can also be effective [41]. Weight loss is recommended alongside other therapies for individuals with obesity.

Complications of tirzepatide

Although the recent trial by Malhotra et al. highlights tirzepatide’s ability to combat OSA, adverse events continue to affect certain subsets of populations. Complications related to tirzepatide therapy are categorized below. 

Cardiovascular and Respiratory Complications

To the best of our knowledge, the current literature does not suggest any increased cardiovascular and respiratory risks with tirzepatide [42]. A recent systematic review analyzing the effects of GLP-1 RAs and tirzepatide on the odds of stroke and stroke types demonstrated decreased major adverse cardiovascular events, all-cause mortality, and cardiovascular mortality in T2DM patients, with no difference between GLP-1 and tirzepatide against placebo [43,44]. For respiratory benefits, tirzepatide has been demonstrated to inhibit aeroallergen-induced allergic airway in a mouse model of asthma [45]. 

Gastrointestinal Complications

The most common adverse events have been gastrointestinal, with nausea, diarrhea, decreased appetite, and vomiting demonstrating increased rates compared to glargine in T2DM patients [46]. Meanwhile, in adults with obesity without T2DM, tirzepatide demonstrates higher significant odds of nausea, vomiting, diarrhea, decreased appetite, eructation, and dyspepsia compared to placebo [47]. Furthermore, studies have documented adverse events such as pancreatitis and cholecystitis. Analyses from Zeng et al. show that there is not a significantly increased relative risk for developing pancreatitis with tirzepatide therapy, though tirzepatide was significantly associated with a relative risk of 1.97 for the development of biliary disease [48]. Additionally, a systematic review by Mishra et al. supports findings by Zeng et al. of pancreatitis cases not posing a large safety risk as the pooled portions of acute pancreatitis cases for the 5 mg, 10 mg, and 15 mg regimens range from 32% to 39% [49]. 

*Renal* *Complications*

Many have regarded tirzepatide for its renal protective effects [50,51] and convenience for lack of necessary renal adjustment [52], though Eli Lilly and Company do warn of the possibility of acute kidney injury. With gastrointestinal events being the most commonly reported adverse events, acute kidney injury may present secondary to volume losses and dehydration [53,54]. 

*Endocrine* *Complications*

Hypoglycemic events have been reported to commonly affect those patients concurrently taking sulfonylureas across all BMI categories in T2DM patients [55]. Additionally, analyses of the SURPASS-3 and SURPASS-4 trials demonstrate that T2DM patients who are currently using sulfonylureas have increased incidences of hypoglycemic events when adjunctive tirzepatide or glargine is added to their regimen [56]. Hypoglycemic events have been noted to not demonstrate a dose escalation pattern, in which rates of hypoglycemia defined as less than 70 mg/dL range from 17.4% to 22.6% and less than 54 mg/dL range from 3.3% to 3.6% if provided the 5 mg, 10 mg, or 15 mg dosing regimen of tirzepatide [49]. 

Another feared endocrinologic complication of tirzepatide therapy includes the development of a thyroid C-cell tumor evidenced by a two-year carcinogenicity study in rats which received an injection of tirzepatide twice weekly. Therefore, tirzepatide is not recommended for patients who have a family history of medullary thyroid cancer or multiple endocrine neoplasia type 2 syndrome [53,57].

Neurologic Complications

There are emerging case reports exploring the association between tirzepatide weight loss and the development of foot drop secondary to peroneal nerve neuropathy [58]. This phenomenon, termed slimmer’s paralysis in 1984, has commonly been associated with weight loss regimens, such as bariatric surgery, that cause a great reduction in weight [59]. 

Dermatologic Complications

The rates of hypersensitivity reactions have been demonstrated to be similar regardless of antidrug antibody status, though those with positive antidrug antibody status have reported non-severe injection site reactions more often [60]. Furthermore, there is a greater odds ratio of experiencing alopecia compared to placebo in those without T2DM [47]. 

Tirzepatide off-label uses and potential indications

Tirzepatide, primarily approved for the treatment of T2DM, has also gained attention for its off-label effects and potential uses beyond its FDA-approved indications. Beyond its weight loss benefits, tirzepatide may offer additional therapeutic effects pertinent to OSA through its metabolic and anti-inflammatory actions. OSA is often accompanied by metabolic dysregulation and systemic inflammation, both of which could be modulated by tirzepatide [61]. By reducing markers of oxidative stress, tirzepatide might indirectly alleviate some of the pathophysiological processes contributing to OSA [62]. Preliminary data indicate that patients treated with tirzepatide exhibit reduced levels of inflammatory markers and improved insulin sensitivity, which could translate to better overall respiratory function and sleep quality [63].

Moreover, tirzepatide's off-label uses extend to cardiovascular health. OSA can lead to hypertension, arrhythmias, and other cardiovascular complications, partly due to intermittent hypoxia and sympathetic nervous system activation [64]. Tirzepatide has been shown to significantly improve cardiovascular outcomes by lowering blood pressure and improving lipid profiles [65]. Additionally, increased adiponectin release and insulin resistance amelioration also contribute to a reduced risk of cardiovascular disease [66]. By improving cardiovascular risk factors, tirzepatide may offer protective effects against conditions such as hypertension, atherosclerosis, and heart failure, which often coexist with metabolic disorders [67]. These cardiovascular improvements may further contribute to the overall management of OSA, highlighting tirzepatide's multifaceted off-label potential in enhancing the quality of life and health outcomes for patients suffering from this prevalent sleep disorder [68]. A post hoc analysis of the SUSTAIN-6 and meta-analysis from the STEP trials found that semaglutide reduced cardiovascular events in patients without established cardiovascular disease. Given tirzepatide’s ability to induce even greater weight loss compared to semaglutide, extrapolated data suggests that it can be an alternative for this purpose as well [69]. 

Another promising off-label use of tirzepatide is its potential role in the management of non-alcoholic fatty liver disease (NAFLD) and non-alcoholic steatohepatitis (NASH). NAFLD and NASH are commonly associated with insulin resistance and metabolic syndrome, conditions which tirzepatide has shown efficacy in addressing [70]. Given the strong association between NAFLD, obesity, and T2DM, tirzepatide’s significant effects on weight loss and improved insulin sensitivity may also contribute to decreasing hepatic fat content, suggesting a promising role in the management of NAFLD [71]. Preliminary studies suggest that tirzepatide can improve hepatic inflammation and reverse fibrosis, offering a potential therapeutic avenue for these liver conditions [72,73]. Given the limited treatment options currently available for NAFLD and NASH, tirzepatide's emerging role could significantly impact patient outcomes, highlighting the need for further research to confirm these benefits and establish appropriate clinical guidelines for its use in these contexts.

The recent FLOW trial using semaglutide in patients with chronic kidney disease found significant decreases in composite major kidney disease events (HR 0.76, 95% CI: 0.66-0.88, p=0.0003) [74]. In a post hoc analysis of SURPASS-4, tirzepatide demonstrated reduced albuminuria and total estimated glomerular filtration rate (eGFR) slopes and nearly decreased the risk of eGFR decline of over 40%, renal death, kidney failure, or new-onset macroalbuminuria by nearly 50%. Tirzepatide also induced decreased eGFR slopes for patients with normoalbuminuria or with an eGFR of >60mL/min/1.73 m^2^ [75]. Thus, tirzepatide’s potential kidney protective effects warrant further research as well. 

More recently, a real-world evidence study using a social media platform included 153 participants (on semaglutide or tirzepatide), among 1580 alcohol-related posts, 71% reported a decrease in cravings, desire to drink, self-reported intake of alcohol, drinks per drinking episode, binge drinking odds, and alcohol use disorders identification test scores (AUDIT) [76]. 

Overall, these emerging findings underscore the promising potential of tirzepatide's off-label applications, warranting further research to further elucidate its efficacy and safety as an expanded therapeutic agent that could benefit a range of metabolic and inflammatory conditions.

Future directions and limitations

The landscape of GLP-1 RA usage will continue to grow and its indications appear to be rapidly evolving beyond obesity and T2DM management. Although this preliminary data suggests the efficacy of tirzepatide in improving OSA symptoms, further large-study RCTs, cohort studies, and real-world evidence studies at longer-term follow-ups are required to confirm these effects. It would also be important to assess if these effects are also seen in patients without obesity.

Despite the promising findings, only two studies investigated tirzepatide for OSA [10,12]. The small number of studies prevents a meta-analysis to pool effect estimates. Additionally, these findings may not be generalizable across other patient populations, subgroups, clinical settings, and OSA severity. 

## Conclusions

OSA is a sleep disorder that can have detrimental effects on the health and quality of life of individuals. Excess weight has been identified as a leading cause of OSA, affecting airflow. Novel treatments have evolved to include prescription medications that may also address the symptoms of and treat OSA. Tirzepatide has been shown to greatly improve glycemic control, reduce substantial weight, and improve digestive and cardiovascular health. At present, the two SURMOUNT-OSA phase 3 trials compared the efficacy of tirzepatide against placebo and have shown that tirzepatide was able to significantly decrease body weight, AHI, PROMIS-SRI, and PROMIS-SD, showing potential as a treatment for OSA.
